# Analysis of non-steroidal anti-inflammatory drug use in hospitalized patients and perception of their risk

**DOI:** 10.2478/intox-2013-0022

**Published:** 2013-09

**Authors:** Zoltán Varga, Milan Kriška, Viera Kristová, Miriam Petrová

**Affiliations:** Department of Pharmacology and Clinical Pharmacology, Faculty of Medicine, Comenius University, Bratislava, Sasinkova 4, 811 08 Bratislava, Slovak Republic

**Keywords:** non-steroidal anti-inflammatory drugs, adverse effect, cardiovascular risk

## Abstract

Non-steroidal anti-inflammatory drugs (NSAIDs) belong to the most widely prescribed and used pharmacological agents worldwide. Data gathered in the last decade show increased incidence of thrombotic events during NSAID administration. Analysis of NSAID usage and assessment of risk for development of cardiovascular adverse effects is needed for improving patient safety. For limiting the impact of adverse effects on the health of patients, NSAID users should be informed about the possible adverse effects and their symptoms to ensure early detection and treatment discontinuation. In the presented study, we retrospectively analyzed the administration of NSAIDs in a group of patients (n=428) in need of analgesic treatment hospitalized at a department of internal medicine. Factors increasing the risk for cardiovascular adverse effects were also investigated. A separate questionnaire study was conducted to gather information concerning the knowledge of hospitalized NSAID users (n=251) about adverse effects of the medication used. For purpose of comparison, we conducted a similar study in a group of 234 random respondents from a shopping center. Data were evaluated using descriptive statistics, Student′s t-test and chi-squared test. Our results suggest that the majority of patients treated with NSAIDs have factors indicating increased risk of development of adverse effects, most commonly arterial hypertension (58.2% of patients). The results of our questionnaire study show limited knowledge of NSAID users about the risk of the therapy. Nearly half of the respondents were unaware of any adverse effects. We consider as alarming that only a limited number of respondents were informed by their physician or pharmacist about the possible risks of treatment. In conclusion, we found that hospitalized NSAID users often have a history of diseases predisposing to the development of cardiovascular adverse effects of NSAIDs. Despite this, their knowledge about the risk of treatment is insufficient.

## Introduction

Non-steroidal anti-inflammatory drugs (NSAIDs) are drugs with high potential for causing drug-induced damage. Nevertheless, they are among the most commonly prescribed and used drugs worldwide. The risk of adverse effects (AEs) affecting the gastrointestinal tract (Douthwaite, 1938) and the kidneys (Walsh & Venuto, 1979) were documented a long time ago. The same holds true for the possibility of increased blood pressure (Staessen *et al.*, 1983) and worsened chronic heart failure (Cannon, 1986) during NSAID administration. In the last decade, there have been reports of an increased incidence of myocardial infarction and other thrombotic events during treatment with selective cyclooxygenase-2 inhibitors and nonselective NSAIDs (Mukherjee, 2002 Bresalier *et al.*, 2005; Trelle *et al.*, 2011; McGettigan & Henry, 2011).

For improved safety of NSAID treatment it is necessary: a) to analyze the use of NSAIDs in patients and to evaluate the risk of AEs and b) to determine and improve the level of user awareness of the potential risks of therapy. In the presented study, we retrospectively analyzed the administration of NSAIDs in a group of patients in need of analgesic treatment, who were hospitalized at a department of internal medicine. The presence of factors increasing the risk for cardiovascular adverse effects was also investigated. A separate questionnaire study was conducted to gather information concerning the knowledge of hospitalized NSAID users about adverse effects of the medication used.

## Methods

Data for our retrospective study were extracted from medical records of patients hospitalized at a department of internal medicine in a county hospital from January 1, 2009 till December 31, 2012. All 428 patients using NSAIDs for a minimum of 4 consecutive days were enrolled.

Our questionnaire contained 16 questions (6 close ended, 7 semi-open-ended, 1 open-ended and 2 visual analogue scales) and was administered face to face. The questionnaire was filled by 251 patients using NSAIDs during hospitalization in a county hospital (study group H). The response rate was over 95%. For purpose of comparison, the same questionnaire was administered to 234 respondents randomly approached in a shopping center (study group SC).

Data were evaluated using descriptive statistics, Student′s t-test and chi-squared test. A *p*-value under 0.05 was considered significant. Results are shown as average ± standard deviation (SD).

Our studies were approved by the authorities of the hospital. All data were gathered in accordance with applicable laws of the Slovak Republic.

## Results

The study group of our retrospective study included 428 patients. A marked predominance of women over men was recorded (271 vs. 157). The average age of the patients was 63.2±16.4 years.

Metamizole was the most commonly administered analgesic in the study group. From traditional NSAIDs, diclofenac was used most often (Table 1). The presence of selected diagnoses in the case histories of patients which can increase the risk of cardiovascular AEs is shown in Table 2. Arterial hypertension was present in nearly 60% of enrolled patients, all of them were treated with antihypertensive drugs. The most widely used antihypertensives were inhibitors of angiotensin converting enzyme (65.1% of hypertensive patients) and beta-blockers (67.5% of hypertensive patients).

**Table 1:** NSAIDs and other non-opioid analgesics used by the patients.

Drug	Number of patients	Percentage of the study group
Metamizole	281	65.7%
Diclofenac	80	18.7%
Indomethacin	72	16.8%
Ketoprofen	68	15.9%
Meloxicam	32	7.5%
Nimesulide	27	6.3%
Paracetamol/acetaminophen*	22	5.1%
Ibuprofen	21	4.9%
Acetylsalicylic acid, 500 mg	10	2.3%
Celecoxib	4	0.9%

* Analgesic-antipyretic.

**Table 2:** Selected diagnoses in the case histories of patients possibly increasing the risk of cardiovascular adverse effects (AE).

Diagnoses increasing the risk of cardiovascular AEs	Number of patients	Percentage of the study group
Arterial hypertension	249	58.2%
Chronic heart failure	184	43.0%
Ischemic heart disease	176	41.1%
Diabetes mellitus	119	27.8%
History of stroke	32	7.5%
Chronic kidney disease	31	7.2%
History of a thrombotic event*	21	4.9%

* Acute myocardial infarction, thrombosis of vena portae and mesenteric veins, thrombosis of vena tibialis posterior, pulmonary embolism

A total of 485 respondents were enrolled in the questionnaire study, of whom 251 filled the questionnaire during hospitalization, 234 were approached in a shopping center. The distribution of gender in the groups was similar. Nearly 60% of respondents were women (H: W–150 (58.1%), M–101; SC: W–136 (59.8%), M–98). In the group of hospitalized patients, we found a predominance of older respondents (60.9±13.8). The average age of the respondents approached in the shopping center was markedly lower (32.4±9.1).

Perception of risk of NSAIDs was determined with a visual analogue scale, where the value of 1 represented the safest and the value of 10 the most dangerous drugs on the market. Hospitalized respondents estimated the risk of NSAIDs to 3.8±1.9. Respondents from the shopping center perceived NSAIDs as significantly more dangerous drugs – 4.7±1.8 (*p<*0.001).

Nearly half of the hospitalized respondents and more than half of the respondents approached in the shopping center were not aware of any adverse effects of NSAIDs. The number of respondents who were unaware of AEs was significantly lower in the group of hospitalized (*p=*0.019). From the possible AEs of NSAIDs, gastric damage was mentioned most often in both groups (Tables 3 and 4).

**Table 3:** Adverse effects of NSAIDs known by the respondents (H).

Adverse effects known by the respondents	Number of respondents	Percentage of the study group
Gastric damage	67	26.7%
Liver damage	63	25.1%
Kidney damage	25	10.0%
Cardiovascular	20	8.0%
Other	46	18.3%
Doesn′t know any	116	46.2%

**Table 4:** Adverse effects of NSAIDs known by the respondents (SC).

Adverse effects known by the respondents	Number of respondents	Percentage of the study group
Gastric damage	74	31.2%
Liver damage	41	17.5%
CNS damage	25	10.7%
Cardiovascular	19	8.1%
Other	34	14.5%
Doesn′t know any	133	56.8%

The physician prescribing the drug or the pharmacist had informed about the risk of drug-induced injury just in 31.1% of our hospitalized respondents and in 41.0% of respondents from the shopping center. The difference between the groups was not significant. Respondents were often informed only if they actively asked about possible AEs. Just a small number of respondents was informed without a direct question (H: 40 – 15.9% of the group, SC: 53 – 22.6% of the group).

## Discussion

### Clinical study

The most commonly administered analgesic was metamizole. The reasons for its frequent use might have been its relatively good analgesic effect, spasmolytic effect and low gastro- and nephrotoxicity. On the other hand, the risk of leukopenia during administration of metamizole has been extensively documented. The incidence of agranulocytosis after a week of treatment is around 1.1:1 000 000 (Arellano & Sacristan, 1990). Our data about prescription of various NSAIDs were in line with the findings of other authors. The only exception was ibuprofen, which was administered less frequently than in previously published studies (Hudec *et al.*, 2008).

The presence of certain diagnoses in the case histories of patients markedly increases the risk of cardiovascular AEs and is thus considered to be an indicator of increased cardiovascular risk of NSAIDs. More than half of the study group had at least one of these diagnoses present, most commonly arterial hypertension. Every hypertensive patient was treated with antihypertensive medication. Our results are in line with the results of Adams *et al.* (2011) who found that 60.8% of NSAID users have arterial hypertension and with the results of Lanas (2011) who documented antihypertensive pharmacotherapy in 57.6% of NSAID users. The most widely used antihypertensive drugs in our study group were drugs with high risk of decrease in antihypertensive effect when co-administered with NSAIDs (Johnson *et al.*, 1994).

Chronic heart failure, ischemic heart disease and diabetes mellitus were also frequently present in the case histories of patients enrolled in our study. Our findings suggest a relatively high risk of cardiovascular AEs in NSAID users hospitalized in a department of internal medicine of a county hospital in Slovakia.

### Questionnaire study

Hospitalized respondents perceived NSAIDs as significantly safer drugs than did respondents from the shopping center. It is likely that due to their higher age and comorbidities, hospitalized respondents are using more drugs regularly than the randomly selected, significantly younger people from a shopping center. It is possible that daily use of a high number of drugs could have a negative effect on the perception of drug risk in some patients as taking drugs has become part of their daily routine. We can′t rule out the possibility that long term treatment of severe heart, oncologic or other diseases could decrease the perception of risk of “ordinary analgesics”. A positive finding is that the perception of risk was higher in both of groups than the perception of risk in NSAID users documented by an earlier study from Ireland (2.1 [0.7–4.9]) (Cullen *et al.*, 2006).

Our results indicate insufficient awareness of NSAID users about possible AEs. We consider as particularly disturbing that without a direct question from the patient physicians and pharmacists inform only every fifth person about AEs. This situation exposes users of NSAIDs to a particular hazard. The danger caused by inadequate awareness can be illustrated with the example of one respondent from the shopping center who developed a peptic ulcer while using NSAIDs and then tried to treat the pain caused by the ulcer with increased doses of the analgesic.

In conclusion, we found that patients using NSAIDs during hospitalization at a department of internal medicine are of higher age, often with increased risk of cardiovascular AEs of NSAIDs. Despite this, hospitalized users of NSAIDs have insufficient knowledge about the risks of their treatment. The lack of knowledge about possible AEs appears to be caused by the small number of physicians and pharmacists taking an active approach in informing the users of NSAIDs about the possibility of drug-related damage.
